# Fluorenonophane chloro­benzene solvate: mol­ecular and crystal structures

**DOI:** 10.1107/S2056989021011865

**Published:** 2021-11-12

**Authors:** Viktoriya V. Dyakonenko, Svitlana V. Shishkina, Tatiana Yu. Bogashchenko, Alexander Yu. Lyapunov, Tatiana I. Kirichenko

**Affiliations:** a SSI ‘Institute for Single Crystals’, National Academy of Sciences of Ukraine, 60 Nauky Ave., Kharkiv 61001, Ukraine; b V. N. Karazin Kharkiv National University, 4 Svobody sq., Kharkiv 61112, Ukraine; c A. V. Bogatsky Physico-Chemical Institute, National Academy of Sciences of Ukraine, 86 Lustdorfskaya doroga, Odesa, Ukraine; d Enamine Ltd., Chervonotkatska Street 78, Kyiv, 02094 , Ukraine; e Taras Shevchenko National University of Kyiv, Volodymyrska Street 60, Kyiv, 01601, Ukraine

**Keywords:** crystal structure, fluorenonophane, hydrogen bonds

## Abstract

The title compound, 1^9^ *H*,7^9^ *H*-3,5,9,11-tetra­oxa-1,7(2,7)-difluorena-4,10(1,3)-dibenzena­cyclo­dodeca­phane-1^9^,7^9^-dione (fluorenonophane), exists as a solvate with chloro­benzene, C_42_H_28_O_6_·C_6_H_5_Cl. The fluorenonophane contains two fluorenone fragments linked by two *m*-substituted benzene fragments. Some decrease in its macrocyclic cavity leads to a stacking inter­action between the tricyclic fluorenone fragments. In the crystal, the fluorenonophane and chloro­benzene mol­ecules are linked by weak C—H⋯π(ring) inter­actions and C—H⋯Cl hydrogen bonds. The Cl atom of chloro­benzene does not form a halogen bond. A Hirshfeld surface analysis and two-dimensional fingerprint plots were used to analyse the inter­molecular contacts found in the crystal structure.

## Abstract 2

The mol­ecular and crystal structures of the chloro­benzene solvate of fluorenonophane have been studied using X-ray diffraction analysis. The fluorenonophane contains two fluorenone fragments linked by two *m*-substituted benzene fragments. Some decrease in its macrocyclic cavity leads to a stacking inter­action between the tricyclic fluorenone fragments. In the crystal, the fluorenonophane and chloro­benzene mol­ecules are linked by weak C—H⋯π(ring) inter­actions and C—H⋯Cl hydrogen bonds.

## Chemical context

Discovered at the end of the last century, the ability of cyclo­phanes to form inclusion complexes makes them the central class of synthetic receptors in mol­ecular recognition processes (Diederich, 1991 ▸). Particular attention has been paid to the possibility of cationic cyclo­phanes with box geometries being involved in strong donor–acceptor inter­actions leading to the formation of ‘guest–host’ complexes with different guests (Dale *et al.*, 2016 ▸; Barnes *et al.*, 2013 ▸; Gong *et al.*, 2010 ▸). Previously we have obtained fluorenonophane **1** with two fluorenone fragments linked by rigid xylyl groups (Lukyanenko *et al.*, 2003 ▸; Simonov *et al.*, 2006 ▸). X-ray diffraction analysis of this cyclo­phane revealed the box geometry with an open intra­molecular cavity and the formation of inclusion complexes with DMF and nitro­benzene (Simonov *et al.*, 2006 ▸). The other fluorenonophane obtained by our group, **2**, differs from the previous one in the position of the methyl­ene groups, which are located directly at the benzene fragment in **1** or fluorenone in **2**. Fluorenonophane **2** forms inclusion complexes with chloro­form and bromo­form with a 1:2 stoichiometry. Moreover, C—Cl⋯π and C—Br⋯π halogen bonds (Shishkina *et al.*, 2021 ▸) are present in the complexes. In contrast to cationic cyclo­phanes, there are no charged fragments in fluorenonophanes. Continuing our research in this area, we have obtained fluorenonophane **3** with a different position of attachment of the benzene rings compared to **2** (*m*- and *p*-isomers, respectively) and studied its complexation with chloro­benzene. 
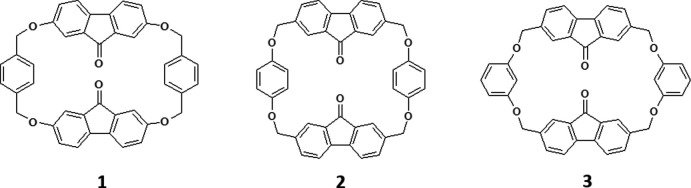


## Structural commentary

**Figure 1:**
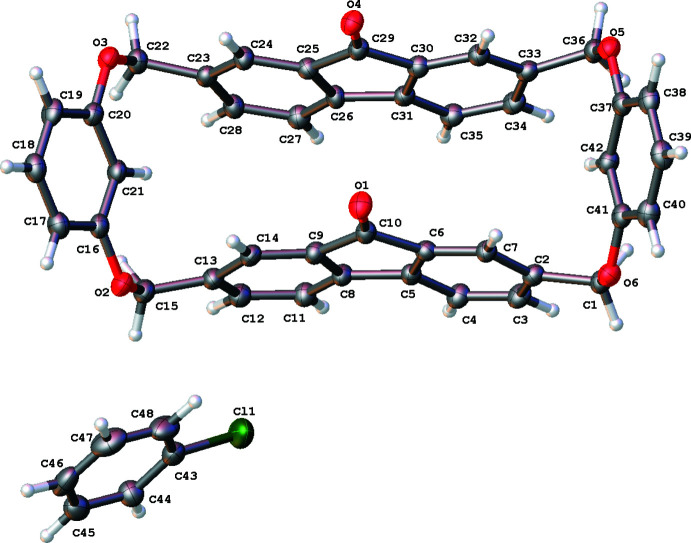
The mol­ecular structure of the title compound showing the atom-labelling scheme. Displacement ellipsoids are drawn at the 50% probability level.

Fluorenonophane **3** was crystallized from chloro­benzene and exists in the crystal as a solvate in a 1:1 ratio rather than as an inclusion complex. Fluorenonophane **3** contains two fluorenone fragments linked by two *m*-substituted benzene fragments (Fig. 1 ▸). The macrocycle **3** has a boat conformation similar to structure **1** [the torsion angles C41—O6—C1—C2, C37—O5—C36—C33, C20—O3—C22—C23, and C16—O2—C15—C13 are −90.6 (4), 78.4 (4), −80.0 (4) and 91.6 (4)°, respectively]. In structure **3**, the fluorenone fragments are oriented in the same directions (*cis*-orientation) while the orientation of these fragments is *trans* in structures **1** and **2**. *meta*-Substitution of the two benzene fragments results in a smaller macrocycle cavity as compared to fluorenonophanes **1** and **2** with *para*-substituted benzene fragments. As a result, the two fluorenones are slightly bowed inwards [the dihedral angle between C2–C7 and C8-C14 benzene rings is 12.51 (18)° in one fluorenone while the dihedral angle between the C31–C35 and C23–C28 benzene rings is 9.64 (18)° in the other fluorenone). This can be explained by a π-stacking inter­action between the C10=O1 carbonyl group and the C25/C26/C31/C30/C29 fluorenone ring [centroid *Cg*2, with O1⋯*Cg*2 = 3.469 (3) Å, C10⋯*Cg*2 = 3.492 (4) Å, C10=O1⋯*Cg*2 = 81.1 (2)°]. In contrast to structures **1** and **2**, the macrocycle in structure **3** does not contain any mol­ecules inside its cavity. Therefore, the structure under study is a chloro­benzene solvate of fluorenonophane.

## Supra­molecular features

**Figure 2:**
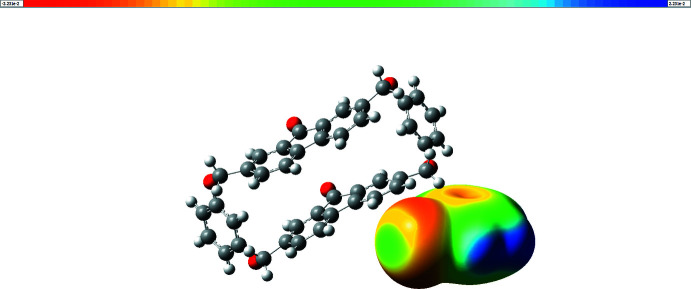
Electrostatic potential map of the chloro­benzene mol­ecule in **3** calculated by the B3LYP/6–311 G(d,p) method.

In the crystal, the fluorenonophane and chloro­benzene mol­ecules are linked to each other by weak C46—H46⋯O6 and C18—H18⋯Cl1 hydrogen bonds while the fluorenophanes are linked by weak C35—H35⋯O1 hydrogen bonds (Table 1 ▸), forming stepped ribbons. The ribbons are connected by C1—H1*A*⋯*Cg*2 and C22—H22*A*⋯*Cg*1 inter­actions (Table 1 ▸) to give the final three-dimensional structure. The halogen atom does not form a halogen bond in the structure of **3**, in contrast to the supra­molecular complexes studied earlier (Shishkina *et al.*, 2021 ▸). The electrostatic potential for chloro­benzene was calculated using the B3LYP/6–311 G(d,p) method. An area with a positive charge (*σ*-hole) was not found in the electrostatic potential map around the halogen atom (Fig. 2 ▸). The highest electrostatic potential at the chlorine atom is −0.08 eV. This fact can explain the absence of halogen bonds in the structure of **3**.

## Hirshfeld surface analysis

**Figure 3:**
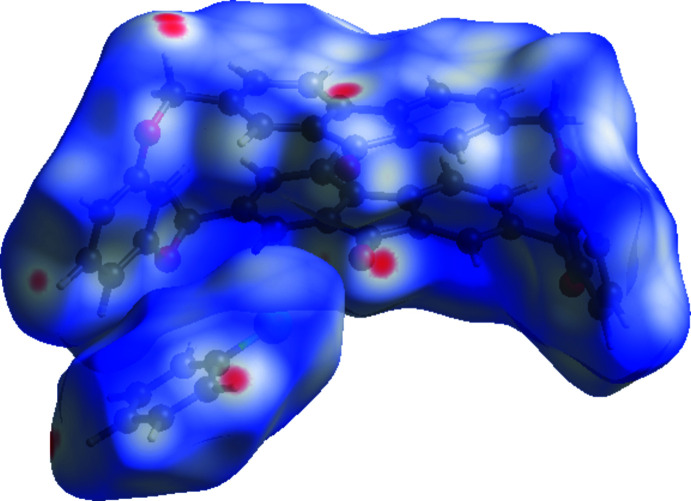
Hirshfeld surface mapped over *d*_norm_ showing the conformation of the fluorenonophane and chloro­benzene mol­ecules.

*Crystal Explorer 17.5* (Turner *et al.*, 2017 ▸) was used to analyze inter­actions in the crystal. Mol­ecular Hirshfeld surfaces mapped over *d*_norm_ with a standard (high) surface resolution and a fixed colour scale of −0.134 (red) to 1.206 (blue) were generated separately (Fig. 3 ▸) for the fluorenonophane and chloro­benzene mol­ecules. The areas in red correspond to contacts that are shorter than the sum of the van der Waals radii of the closest atoms. Thus, the red spots at some hydrogen atoms and at the carbonyl oxygen atom as well as in the area of the five-membered ring indicate the existence of short C—H⋯O and C—H⋯π(ring) contacts.

**Figure 4:**
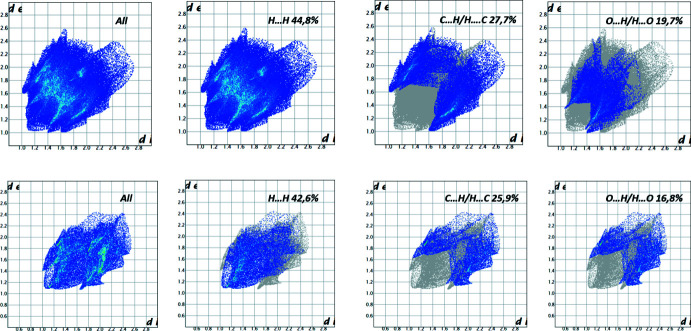
The two-dimensional fingerprint plots for fluorenonophane **3** (top) and chloro­benzene (bottom).

To evaluate the contribution of the short contacts of different types to the total Hirshfeld surface, two-dimensional fingerprint plots for the fluorenonophane and chloro­benzene mol­ecules were generated (Fig. 4 ▸). The contribution from the C⋯H/H⋯C contacts corresponding to the C—H⋯π(ring) inter­actions are represented by a pair of sharp spikes (27.7% and 25.9% for fluorenonophane and chloro­benzene, respectively). Analysis of the fingerprint plots also showed a significant contribution from O⋯H/H⋯O contacts (19.7%) associated with the C—H⋯O hydrogen bonds.

## Database survey

A search of the Cambridge Structural Database (CSD, Version 5.42, update of November 2020; Groom *et al.*, 2016 ▸) for cyclo­phanes containing fluorenone and benzene fragments yielded two hits: two structures with fluorenone fragments linked by rigid xylyl groups (CCDC 263272 and CCDC 263273; Simonov *et al.*, 2006 ▸). Recently, two more structures with fluorenonophanes linked by *para*-substituted benzene fragments were published (CCDC 647971 and CCDC 2098245; Shishkina *et al.*, 2021 ▸). The structures found are characterized by a larger macrocyclic cavity compared to that in fluorenonophane **3**.

## Synthesis and crystallization

**Figure 5:**
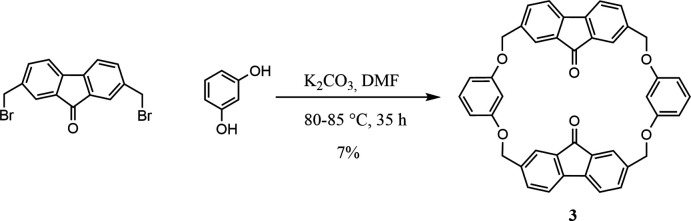
The synthesis of fluorenonophane **3**

A solution of 1.75 g (4.78 mmol) of 2,7-bis­(bromo­meth­yl)-9*H*-fluoren-9-one (Haenel *et al.*, 1985 ▸) in 200 mL of anhydrous DMF was added to a mixture of 0.526 g (4.78 mmol) of resorcinol and 3.96 g (28.7 mmol) of K_2_CO_3_ in 270 mL of anhydrous DMF with stirring under nitro­gen for 10 h at 353–358 K. The reaction mixture was stirred at the same temperature for a further 35 h, cooled and filtered (Fig. 5 ▸). The precipitate was washed with DMF and the filtrate was evaporated under reduced pressure. The residue was dissolved in CHCl_3_ and washed with an aqueous sodium carbonate solution (50 mL), then with water (3 × 50 mL) to a neutral pH. After drying over MgSO_4_, the CHCl_3_ was evaporated under reduced pressure. The product was purified by chromatography on silica gel (Acros 0.060 ÷ 1/5), eluent CHCl_3_–EtOH, 500:1. The yield of cyclo­phane **3** was 0.11 g (7.2%), m.p. >573 K, dec. ^1^H NMR (DMSO-*d*_6_), *δ*, p.p.m.: 5.25 *s* (CH_2_, 8H), 6.46–6.56 *m* (H_2_, H_4_, 6H), 7.04 *t* (H_5_, 2H, *J* = 8.1 Hz), 7.18 *s* (H*a*, 4H), 7.57 *m* (H*b*, HH, 8H). MS: FAB, *m*/*z* 628 [*M* + H^+^]. Analysis calculated for C_42_H_28_O_6_: C, 80.24; H, 4.49. Found: C, 80.44; H, 4.76%. Crystals were obtained by crystallization of fluorenonophane **3** from chloro­benzene.

## Refinement

Crystal data, data collection, and structure refinement details are summarized in Table 2 ▸. Carbon-bound H atoms were added in calculated positions with C—H bond lengths of 0.95 Å for C—H, 0.92 Å for CH_2_ and refined as riding atoms with *U*_iso_(H) = 1.2*U*_eq_(C).

## Supplementary Material

Crystal structure: contains datablock(s) I. DOI: 10.1107/S2056989021011865/mw2179sup1.cif

Structure factors: contains datablock(s) I. DOI: 10.1107/S2056989021011865/mw2179Isup2.hkl

CCDC reference: 2121128

Additional supporting information:  crystallographic information; 3D view; checkCIF report

## Figures and Tables

**Table 1:** Hydrogen-bond geometry (Å, °) *Cg*1, *Cg*2 and *Cg*15 are the centroids of the C5/C6/C10/C9/C8, C25/C29/C30/C31/C26 and C43–C48 rings, respectively.

*D*—H⋯*A*	*D*—H	H⋯*A*	*D*⋯*A*	*D*—H⋯*A*
C18—H18⋯Cl1^i^	0.95	2.83	3.547 (4)	133
C35—H35⋯O1^ii^	0.95	2.58	3.491 (5)	161
C46—H46⋯O6^iii^	0.95	2.55	3.418 (5)	152
C1—H1*A*⋯*Cg*2^iv^	0.99	2.95	3.610 (4)	125
C22—H22*A*⋯*Cg*1^v^	0.99	2.73	3.711 (4)	170
C36—H36*B*⋯*Cg*15^vi^	0.99	2.84	3.713 (4)	148

Symmetry codes: (i) x+1, y+1, z; (ii) x-1, y, z; (iii) x+1, y+1, z+1; (iv) x, y-1, z; (v) x, y+1, z; (vi) x-1, y, z-1.

**Table 2:** Experimental details

Crystal data	
Chemical formula	C_42_H_28_O_6_·C_6_H_5_Cl
*M*_r_	741.19
Crystal system, space group	Triclinic, *P*1
Temperature (K)	100
*a*, *b*, *c* (Å)	6.2278 (6), 9.6965 (8), 14.9822 (13)
α, β, γ (°)	105.288 (8), 97.126 (7), 96.919 (7)
*V* (Å^3^)	854.83 (13)
*Z*	1
Radiation type	Mo *K*α
μ (mm^−1^)	0.17
Crystal size (mm)	0.6 × 0.4 × 0.2

Data collection	
Diffractometer	Xcalibur, Sapphire3
Absorption correction	Multi-scan (*CrysAlis PRO*; Rigaku OD, 2018 ▸)
*T*_min_, *T*_max_	0.846, 1.000
No. of measured, independent and observed [*I* > 2σ(*I*)] reflections	8226, 7191, 5307
*R*_int_	0.028
(sin θ/λ)_max_ (Å^−1^)	0.808

Refinement	
*R*[*F* ^2^ > 2σ(*F* ^2^)], *wR*(*F* ^2^), *S*	0.064, 0.171, 1.03
No. of reflections	7191
No. of parameters	496
No. of restraints	3
H-atom treatment	H-atom parameters constrained
Δρ_max_, Δρ_min_ (e Å^−3^)	0.79, −0.42
Absolute structure	Flack *x* determined using 564 quotients [(*I* ^+^)−(*I* ^−^)]/[(*I* ^+^)+(*I* ^−^)] (Parsons *et al.*, 2013 ▸)
Absolute structure parameter	0.19 (9)

Computer programs: *CrysAlis PRO* (Rigaku OD, 2018 ▸), *SHELXT* (Sheldrick, 2015*a* ▸), *SHELXL2018/3* (Sheldrick, 2015*b* ▸) and *OLEX2* (Dolomanov *et al.*, 2009 ▸).

## supplementary crystallographic information

### Crystal data

**Table:**

C_42_H_28_O_6_·C_6_H_5_Cl	*Z* = 1
*M**_r_* = 741.19	*F*(000) = 386
Triclinic, *P*1	*D*_x_ = 1.440 Mg m^−^^3^
*a* = 6.2278 (6) Å	Mo *K*α radiation, λ = 0.71073 Å
*b* = 9.6965 (8) Å	Cell parameters from 1987 reflections
*c* = 14.9822 (13) Å	θ = 3.9–33.0°
α = 105.288 (8)°	µ = 0.17 mm^−^^1^
β = 97.126 (7)°	*T* = 100 K
γ = 96.919 (7)°	Block, colourless
*V* = 854.83 (13) Å^3^	0.6 × 0.4 × 0.2 mm

### Data collection

**Table:**

Xcalibur, Sapphire3 diffractometer	7191 independent reflections
Radiation source: fine-focus sealed X-ray tube, Enhance (Mo) X-ray Source	5307 reflections with *I* > 2σ(*I*)
Graphite monochromator	*R*_int_ = 0.028
Detector resolution: 16.1827 pixels mm^-1^	θ_max_ = 35.0°, θ_min_ = 3.0°
ω scans	*h* = −9→8
Absorption correction: multi-scan (CrysAlisPro; Rigaku OD, 2018)	*k* = −7→15
*T*_min_ = 0.846, *T*_max_ = 1.000	*l* = −24→20
8226 measured reflections	

### Refinement

**Table:**

Refinement on *F*^2^	Hydrogen site location: inferred from neighbouring sites
Least-squares matrix: full	H-atom parameters constrained
*R*[*F*^2^ > 2σ(*F*^2^)] = 0.064	*w* = 1/[σ^2^(*F*_o_^2^) + (0.0825*P*)^2^ + 0.017*P*] where *P* = (*F*_o_^2^ + 2*F*_c_^2^)/3
*wR*(*F*^2^) = 0.171	(Δ/σ)_max_ < 0.001
*S* = 1.03	Δρ_max_ = 0.79 e Å^−^^3^
7191 reflections	Δρ_min_ = −0.42 e Å^−^^3^
496 parameters	Absolute structure: Flack *x* determined using 564 quotients [(*I*^+^)-(*I*^-^)]/[(*I*^+^)+(*I*^-^)] (Parsons et al.,2013)
3 restraints	Absolute structure parameter: 0.19 (9)

### Special details

**Table:**

Geometry. All esds (except the esd in the dihedral angle between two l.s. planes) are estimated using the full covariance matrix. The cell esds are taken into account individually in the estimation of esds in distances, angles and torsion angles; correlations between esds in cell parameters are only used when they are defined by crystal symmetry. An approximate (isotropic) treatment of cell esds is used for estimating esds involving l.s. planes.

### Fractional atomic coordinates and isotropic or equivalent isotropic displacement parameters (Å^2^)

**Table:**

	*x*	*y*	*z*	*U*_iso_*/*U*_eq_	
O1	0.3386 (4)	0.2409 (3)	0.30318 (19)	0.0294 (6)	
O2	0.3907 (5)	0.5919 (3)	0.66155 (19)	0.0298 (6)	
O3	0.3627 (5)	0.9250 (3)	0.47327 (18)	0.0265 (5)	
O4	0.3771 (4)	0.5007 (3)	0.14591 (18)	0.0265 (5)	
O5	0.0097 (4)	−0.0028 (3)	−0.12980 (18)	0.0264 (5)	
O6	0.0323 (4)	−0.3103 (3)	0.07559 (18)	0.0259 (5)	
C1	−0.1767 (6)	−0.2869 (4)	0.0994 (3)	0.0245 (7)	
H1A	−0.238920	−0.369009	0.120950	0.029*	
H1B	−0.275117	−0.286764	0.042198	0.029*	
C2	−0.1747 (6)	−0.1482 (4)	0.1741 (2)	0.0239 (7)	
C3	−0.3613 (6)	−0.1340 (4)	0.2162 (3)	0.0266 (7)	
H3	−0.485939	−0.207766	0.193390	0.032*	
C4	−0.3703 (6)	−0.0156 (4)	0.2902 (3)	0.0267 (7)	
H4	−0.498316	−0.007812	0.318459	0.032*	
C5	−0.1889 (6)	0.0909 (4)	0.3217 (2)	0.0222 (6)	
C6	−0.0046 (6)	0.0796 (4)	0.2766 (2)	0.0220 (6)	
C7	0.0060 (6)	−0.0388 (4)	0.2035 (2)	0.0224 (6)	
H7	0.132576	−0.045426	0.174069	0.027*	
C8	−0.1381 (6)	0.2194 (4)	0.4039 (2)	0.0225 (6)	
C9	0.0774 (6)	0.2848 (4)	0.4097 (2)	0.0244 (7)	
C10	0.1658 (6)	0.2076 (4)	0.3267 (2)	0.0231 (7)	
C11	−0.2608 (6)	0.2752 (4)	0.4711 (3)	0.0261 (7)	
H11	−0.410474	0.235645	0.465987	0.031*	
C12	−0.1588 (7)	0.3914 (4)	0.5467 (3)	0.0278 (7)	
H12	−0.242323	0.432694	0.592837	0.033*	
C13	0.0608 (6)	0.4491 (4)	0.5571 (2)	0.0250 (7)	
C14	0.1794 (6)	0.3979 (4)	0.4864 (3)	0.0244 (7)	
H14	0.327603	0.439670	0.490436	0.029*	
C15	0.1603 (7)	0.5576 (4)	0.6484 (3)	0.0290 (8)	
H15A	0.097362	0.647655	0.652418	0.035*	
H15B	0.118770	0.519599	0.700067	0.035*	
C16	0.4764 (6)	0.7076 (4)	0.6331 (2)	0.0251 (7)	
C17	0.6890 (7)	0.7711 (4)	0.6749 (3)	0.0298 (8)	
H17	0.766706	0.736390	0.720920	0.036*	
C18	0.7850 (7)	0.8861 (5)	0.6479 (3)	0.0316 (8)	
H18	0.930083	0.931503	0.676396	0.038*	
C19	0.6746 (6)	0.9367 (4)	0.5802 (3)	0.0297 (8)	
H19	0.743820	1.015419	0.561995	0.036*	
C20	0.4627 (6)	0.8717 (4)	0.5394 (2)	0.0245 (7)	
C21	0.3627 (6)	0.7559 (4)	0.5661 (2)	0.0256 (7)	
H21	0.217055	0.710835	0.538118	0.031*	
C22	0.1320 (6)	0.8832 (4)	0.4482 (3)	0.0260 (7)	
H22A	0.071510	0.954612	0.419840	0.031*	
H22B	0.068110	0.886179	0.505817	0.031*	
C23	0.0619 (6)	0.7348 (4)	0.3806 (2)	0.0231 (7)	
C24	0.1890 (6)	0.6774 (4)	0.3150 (2)	0.0242 (7)	
H24	0.329679	0.727598	0.314978	0.029*	
C25	0.1081 (6)	0.5461 (4)	0.2498 (2)	0.0220 (6)	
C26	−0.0988 (6)	0.4699 (4)	0.2487 (2)	0.0222 (6)	
C27	−0.2240 (6)	0.5237 (4)	0.3154 (3)	0.0260 (7)	
H27	−0.362640	0.471796	0.316539	0.031*	
C28	−0.1408 (6)	0.6572 (4)	0.3816 (2)	0.0250 (7)	
H28	−0.224639	0.695843	0.428392	0.030*	
C29	0.2013 (6)	0.4666 (4)	0.1678 (2)	0.0230 (6)	
C30	0.0310 (6)	0.3407 (4)	0.1173 (2)	0.0236 (7)	
C31	−0.1468 (6)	0.3419 (4)	0.1661 (2)	0.0222 (6)	
C32	0.0253 (6)	0.2392 (4)	0.0333 (2)	0.0232 (6)	
H32	0.148081	0.238534	0.001502	0.028*	
C33	−0.1620 (6)	0.1380 (4)	−0.0043 (3)	0.0243 (7)	
C34	−0.3346 (6)	0.1355 (4)	0.0463 (3)	0.0260 (7)	
H34	−0.459675	0.062432	0.021745	0.031*	
C35	−0.3285 (6)	0.2373 (4)	0.1318 (3)	0.0258 (7)	
H35	−0.447445	0.234509	0.165682	0.031*	
C36	−0.1881 (6)	0.0386 (4)	−0.1026 (2)	0.0261 (7)	
H36A	−0.292015	−0.049858	−0.107536	0.031*	
H36B	−0.253593	0.087410	−0.147047	0.031*	
C37	0.0872 (6)	−0.1085 (4)	−0.0968 (2)	0.0243 (7)	
C38	0.2524 (6)	−0.1682 (4)	−0.1384 (3)	0.0294 (8)	
H38	0.305188	−0.136765	−0.187734	0.035*	
C39	0.3404 (6)	−0.2748 (4)	−0.1071 (3)	0.0296 (8)	
H39	0.454620	−0.316585	−0.135380	0.036*	
C40	0.2643 (6)	−0.3216 (4)	−0.0352 (3)	0.0292 (8)	
H40	0.325432	−0.394801	−0.014078	0.035*	
C41	0.0986 (6)	−0.2601 (4)	0.0050 (2)	0.0250 (7)	
C42	0.0074 (6)	−0.1549 (4)	−0.0254 (3)	0.0246 (7)	
H42	−0.108662	−0.114593	0.002177	0.030*	
Cl1	0.2053 (2)	0.17453 (13)	0.65929 (9)	0.0542 (4)	
C43	0.4128 (7)	0.2615 (4)	0.7525 (3)	0.0306 (8)	
C44	0.3680 (7)	0.2937 (5)	0.8418 (3)	0.0320 (8)	
H44	0.224785	0.265769	0.853168	0.038*	
C45	0.5314 (8)	0.3668 (5)	0.9151 (3)	0.0348 (9)	
H45	0.502082	0.389116	0.977568	0.042*	
C46	0.7375 (8)	0.4077 (5)	0.8978 (3)	0.0406 (10)	
H46	0.850033	0.460205	0.948342	0.049*	
C47	0.7812 (9)	0.3732 (6)	0.8083 (4)	0.0484 (12)	
H47	0.924417	0.401211	0.796899	0.058*	
C48	0.6189 (9)	0.2979 (5)	0.7341 (3)	0.0418 (11)	
H48	0.649317	0.271929	0.671735	0.050*	

### Atomic displacement parameters (Å^2^)

**Table:**

	*U*^11^	*U*^22^	*U*^33^	*U*^12^	*U*^13^	*U*^23^
O1	0.0216 (13)	0.0295 (14)	0.0336 (14)	−0.0023 (10)	0.0080 (10)	0.0040 (11)
O2	0.0340 (15)	0.0216 (12)	0.0304 (13)	−0.0017 (10)	0.0013 (11)	0.0058 (10)
O3	0.0278 (14)	0.0216 (12)	0.0279 (13)	0.0000 (10)	0.0036 (10)	0.0051 (9)
O4	0.0219 (13)	0.0244 (13)	0.0315 (13)	0.0004 (10)	0.0054 (10)	0.0058 (10)
O5	0.0267 (14)	0.0236 (12)	0.0270 (12)	−0.0006 (10)	0.0049 (10)	0.0057 (10)
O6	0.0241 (13)	0.0235 (12)	0.0300 (13)	0.0042 (10)	0.0055 (10)	0.0069 (10)
C1	0.0231 (17)	0.0225 (16)	0.0256 (16)	−0.0001 (12)	0.0047 (12)	0.0043 (12)
C2	0.0216 (17)	0.0219 (16)	0.0274 (17)	0.0009 (12)	0.0033 (13)	0.0072 (12)
C3	0.0193 (16)	0.0246 (17)	0.0348 (18)	−0.0007 (13)	0.0064 (13)	0.0077 (14)
C4	0.0204 (16)	0.0256 (17)	0.0326 (18)	−0.0005 (13)	0.0067 (13)	0.0064 (14)
C5	0.0227 (17)	0.0215 (16)	0.0219 (15)	0.0037 (12)	0.0035 (12)	0.0052 (12)
C6	0.0202 (16)	0.0233 (16)	0.0227 (15)	0.0016 (12)	0.0042 (12)	0.0074 (12)
C7	0.0210 (16)	0.0221 (16)	0.0232 (15)	0.0006 (12)	0.0052 (12)	0.0053 (12)
C8	0.0222 (16)	0.0214 (16)	0.0233 (15)	0.0009 (12)	0.0072 (12)	0.0048 (12)
C9	0.0242 (17)	0.0220 (16)	0.0262 (17)	0.0018 (13)	0.0055 (13)	0.0056 (13)
C10	0.0206 (16)	0.0224 (16)	0.0245 (16)	0.0021 (12)	0.0032 (12)	0.0044 (12)
C11	0.0225 (17)	0.0262 (18)	0.0282 (17)	0.0003 (13)	0.0065 (13)	0.0058 (13)
C12	0.0289 (19)	0.0269 (18)	0.0275 (17)	0.0025 (14)	0.0083 (14)	0.0067 (14)
C13	0.0271 (18)	0.0231 (17)	0.0237 (16)	−0.0005 (13)	0.0044 (13)	0.0064 (13)
C14	0.0258 (18)	0.0199 (16)	0.0263 (16)	0.0007 (13)	0.0040 (13)	0.0061 (12)
C15	0.032 (2)	0.0248 (17)	0.0268 (18)	−0.0030 (14)	0.0045 (14)	0.0051 (14)
C16	0.0253 (17)	0.0234 (16)	0.0229 (16)	0.0005 (13)	0.0049 (13)	0.0009 (12)
C17	0.0252 (18)	0.032 (2)	0.0271 (17)	0.0011 (14)	0.0012 (13)	0.0030 (14)
C18	0.0242 (18)	0.035 (2)	0.0291 (18)	−0.0017 (14)	0.0027 (14)	0.0006 (15)
C19	0.0227 (18)	0.0254 (18)	0.0348 (19)	−0.0057 (13)	0.0051 (14)	0.0019 (14)
C20	0.0242 (17)	0.0224 (16)	0.0252 (16)	0.0018 (12)	0.0055 (12)	0.0041 (12)
C21	0.0254 (17)	0.0224 (16)	0.0243 (16)	−0.0028 (13)	0.0027 (12)	0.0022 (12)
C22	0.0239 (17)	0.0257 (17)	0.0255 (16)	0.0027 (13)	0.0027 (13)	0.0031 (13)
C23	0.0229 (17)	0.0220 (16)	0.0240 (16)	0.0032 (13)	0.0021 (12)	0.0067 (12)
C24	0.0222 (17)	0.0242 (16)	0.0235 (16)	−0.0005 (13)	0.0045 (12)	0.0036 (12)
C25	0.0189 (16)	0.0238 (16)	0.0227 (15)	0.0011 (12)	0.0039 (12)	0.0062 (12)
C26	0.0189 (15)	0.0236 (16)	0.0207 (15)	0.0000 (12)	0.0018 (11)	0.0024 (12)
C27	0.0202 (17)	0.0284 (18)	0.0286 (17)	0.0017 (13)	0.0048 (13)	0.0071 (14)
C28	0.0225 (17)	0.0271 (17)	0.0232 (16)	0.0021 (13)	0.0036 (13)	0.0041 (13)
C29	0.0207 (16)	0.0219 (15)	0.0249 (16)	0.0014 (12)	0.0039 (12)	0.0051 (12)
C30	0.0193 (16)	0.0245 (17)	0.0259 (16)	0.0006 (13)	0.0027 (12)	0.0069 (13)
C31	0.0214 (16)	0.0218 (15)	0.0238 (15)	0.0027 (12)	0.0039 (12)	0.0073 (12)
C32	0.0212 (16)	0.0238 (16)	0.0245 (16)	0.0021 (12)	0.0043 (12)	0.0069 (13)
C33	0.0228 (17)	0.0223 (16)	0.0273 (16)	0.0005 (12)	0.0025 (13)	0.0083 (13)
C34	0.0194 (16)	0.0245 (17)	0.0326 (18)	−0.0022 (12)	0.0013 (13)	0.0092 (14)
C35	0.0196 (16)	0.0274 (17)	0.0302 (17)	−0.0004 (13)	0.0043 (13)	0.0095 (13)
C36	0.0274 (18)	0.0229 (16)	0.0246 (16)	0.0010 (13)	0.0028 (13)	0.0031 (12)
C37	0.0223 (17)	0.0241 (16)	0.0221 (16)	−0.0011 (13)	0.0009 (12)	0.0024 (12)
C38	0.0209 (17)	0.0311 (19)	0.0293 (18)	−0.0044 (14)	0.0036 (13)	0.0008 (14)
C39	0.0199 (17)	0.035 (2)	0.0293 (18)	0.0029 (14)	0.0072 (13)	0.0002 (14)
C40	0.0198 (17)	0.0303 (19)	0.0333 (19)	0.0033 (14)	0.0017 (14)	0.0032 (15)
C41	0.0210 (16)	0.0232 (16)	0.0261 (16)	−0.0021 (12)	0.0025 (12)	0.0024 (13)
C42	0.0220 (17)	0.0218 (16)	0.0278 (16)	0.0012 (12)	0.0051 (12)	0.0036 (12)
Cl1	0.0703 (9)	0.0375 (6)	0.0415 (6)	−0.0101 (5)	−0.0137 (5)	0.0074 (5)
C43	0.035 (2)	0.0242 (18)	0.0315 (19)	0.0030 (15)	0.0041 (15)	0.0075 (14)
C44	0.028 (2)	0.036 (2)	0.0318 (19)	0.0023 (15)	0.0064 (15)	0.0088 (15)
C45	0.040 (2)	0.034 (2)	0.033 (2)	0.0101 (17)	0.0089 (17)	0.0093 (16)
C46	0.037 (2)	0.0231 (19)	0.056 (3)	0.0001 (16)	−0.005 (2)	0.0093 (18)
C47	0.036 (3)	0.042 (3)	0.074 (4)	−0.001 (2)	0.014 (2)	0.028 (3)
C48	0.053 (3)	0.038 (2)	0.042 (2)	0.004 (2)	0.024 (2)	0.0173 (19)

### Geometric parameters (Å, º)

**Table:**

O1—C10	1.207 (4)	C22—H22B	0.9900
O2—C15	1.410 (5)	C22—C23	1.502 (5)
O2—C16	1.374 (5)	C23—C24	1.383 (5)
O3—C20	1.353 (5)	C23—C28	1.393 (5)
O3—C22	1.420 (5)	C24—H24	0.9500
O4—C29	1.214 (4)	C24—C25	1.377 (5)
O5—C36	1.415 (5)	C25—C26	1.402 (5)
O5—C37	1.362 (4)	C25—C29	1.492 (5)
O6—C1	1.420 (4)	C26—C27	1.377 (5)
O6—C41	1.363 (4)	C26—C31	1.473 (5)
C1—H1A	0.9900	C27—H27	0.9500
C1—H1B	0.9900	C27—C28	1.401 (5)
C1—C2	1.504 (5)	C28—H28	0.9500
C2—C3	1.393 (5)	C29—C30	1.479 (5)
C2—C7	1.389 (5)	C30—C31	1.400 (5)
C3—H3	0.9500	C30—C32	1.371 (5)
C3—C4	1.382 (5)	C31—C35	1.370 (5)
C4—H4	0.9500	C32—H32	0.9500
C4—C5	1.376 (5)	C32—C33	1.382 (5)
C5—C6	1.404 (5)	C33—C34	1.392 (5)
C5—C8	1.472 (5)	C33—C36	1.509 (5)
C6—C7	1.377 (5)	C34—H34	0.9500
C6—C10	1.491 (5)	C34—C35	1.389 (5)
C7—H7	0.9500	C35—H35	0.9500
C8—C9	1.396 (5)	C36—H36A	0.9900
C8—C11	1.376 (5)	C36—H36B	0.9900
C9—C10	1.479 (5)	C37—C38	1.377 (5)
C9—C14	1.383 (5)	C37—C42	1.388 (5)
C11—H11	0.9500	C38—H38	0.9500
C11—C12	1.390 (5)	C38—C39	1.384 (6)
C12—H12	0.9500	C39—H39	0.9500
C12—C13	1.387 (5)	C39—C40	1.387 (6)
C13—C14	1.386 (5)	C40—H40	0.9500
C13—C15	1.495 (5)	C40—C41	1.377 (5)
C14—H14	0.9500	C41—C42	1.375 (5)
C15—H15A	0.9900	C42—H42	0.9500
C15—H15B	0.9900	Cl1—C43	1.732 (4)
C16—C17	1.385 (5)	C43—C44	1.362 (5)
C16—C21	1.372 (5)	C43—C48	1.371 (6)
C17—H17	0.9500	C44—H44	0.9500
C17—C18	1.378 (6)	C44—C45	1.372 (6)
C18—H18	0.9500	C45—H45	0.9500
C18—C19	1.383 (6)	C45—C46	1.373 (7)
C19—H19	0.9500	C46—H46	0.9500
C19—C20	1.382 (5)	C46—C47	1.364 (7)
C20—C21	1.392 (5)	C47—H47	0.9500
C21—H21	0.9500	C47—C48	1.381 (7)
C22—H22A	0.9900	C48—H48	0.9500
			
C16—O2—C15	117.1 (3)	C24—C23—C28	119.8 (3)
C20—O3—C22	116.5 (3)	C28—C23—C22	118.7 (3)
C37—O5—C36	116.8 (3)	C23—C24—H24	120.6
C41—O6—C1	118.1 (3)	C25—C24—C23	118.8 (3)
O6—C1—H1A	108.6	C25—C24—H24	120.6
O6—C1—H1B	108.6	C24—C25—C26	121.5 (3)
O6—C1—C2	114.5 (3)	C24—C25—C29	129.7 (3)
H1A—C1—H1B	107.6	C26—C25—C29	108.5 (3)
C2—C1—H1A	108.6	C25—C26—C31	108.6 (3)
C2—C1—H1B	108.6	C27—C26—C25	120.2 (3)
C3—C2—C1	117.3 (3)	C27—C26—C31	131.1 (3)
C7—C2—C1	122.5 (3)	C26—C27—H27	121.0
C7—C2—C3	120.2 (3)	C26—C27—C28	118.0 (3)
C2—C3—H3	119.1	C28—C27—H27	121.0
C4—C3—C2	121.9 (3)	C23—C28—C27	121.6 (3)
C4—C3—H3	119.1	C23—C28—H28	119.2
C3—C4—H4	120.9	C27—C28—H28	119.2
C5—C4—C3	118.3 (3)	O4—C29—C25	127.1 (3)
C5—C4—H4	120.9	O4—C29—C30	127.5 (3)
C4—C5—C6	119.9 (3)	C30—C29—C25	105.3 (3)
C4—C5—C8	131.2 (3)	C31—C30—C29	109.1 (3)
C6—C5—C8	108.7 (3)	C32—C30—C29	129.5 (3)
C5—C6—C10	107.9 (3)	C32—C30—C31	121.3 (3)
C7—C6—C5	122.1 (3)	C30—C31—C26	108.4 (3)
C7—C6—C10	129.9 (3)	C35—C31—C26	131.4 (3)
C2—C7—H7	121.2	C35—C31—C30	120.1 (3)
C6—C7—C2	117.7 (3)	C30—C32—H32	120.5
C6—C7—H7	121.2	C30—C32—C33	119.0 (3)
C9—C8—C5	108.5 (3)	C33—C32—H32	120.5
C11—C8—C5	131.3 (3)	C32—C33—C34	119.5 (3)
C11—C8—C9	120.1 (3)	C32—C33—C36	120.7 (3)
C8—C9—C10	108.7 (3)	C34—C33—C36	119.7 (3)
C14—C9—C8	121.4 (3)	C33—C34—H34	119.2
C14—C9—C10	129.8 (3)	C35—C34—C33	121.6 (3)
O1—C10—C6	126.6 (3)	C35—C34—H34	119.2
O1—C10—C9	127.7 (3)	C31—C35—C34	118.4 (3)
C9—C10—C6	105.7 (3)	C31—C35—H35	120.8
C8—C11—H11	121.1	C34—C35—H35	120.8
C8—C11—C12	117.9 (3)	O5—C36—C33	114.3 (3)
C12—C11—H11	121.1	O5—C36—H36A	108.7
C11—C12—H12	118.9	O5—C36—H36B	108.7
C13—C12—C11	122.3 (4)	C33—C36—H36A	108.7
C13—C12—H12	118.9	C33—C36—H36B	108.7
C12—C13—C15	117.1 (3)	H36A—C36—H36B	107.6
C14—C13—C12	119.3 (3)	O5—C37—C38	116.1 (3)
C14—C13—C15	123.4 (3)	O5—C37—C42	123.0 (3)
C9—C14—C13	118.6 (3)	C38—C37—C42	120.9 (4)
C9—C14—H14	120.7	C37—C38—H38	120.5
C13—C14—H14	120.7	C37—C38—C39	118.9 (3)
O2—C15—C13	114.4 (3)	C39—C38—H38	120.5
O2—C15—H15A	108.7	C38—C39—H39	119.5
O2—C15—H15B	108.7	C38—C39—C40	121.0 (4)
C13—C15—H15A	108.7	C40—C39—H39	119.5
C13—C15—H15B	108.7	C39—C40—H40	120.6
H15A—C15—H15B	107.6	C41—C40—C39	118.8 (4)
O2—C16—C17	115.7 (3)	C41—C40—H40	120.6
C21—C16—O2	122.8 (3)	O6—C41—C40	115.9 (3)
C21—C16—C17	121.5 (3)	O6—C41—C42	122.9 (3)
C16—C17—H17	120.9	C42—C41—C40	121.2 (3)
C18—C17—C16	118.3 (4)	C37—C42—H42	120.5
C18—C17—H17	120.9	C41—C42—C37	119.1 (3)
C17—C18—H18	119.3	C41—C42—H42	120.5
C17—C18—C19	121.5 (4)	C44—C43—Cl1	119.8 (3)
C19—C18—H18	119.3	C44—C43—C48	121.6 (4)
C18—C19—H19	120.3	C48—C43—Cl1	118.6 (3)
C20—C19—C18	119.3 (3)	C43—C44—H44	120.3
C20—C19—H19	120.3	C43—C44—C45	119.5 (4)
O3—C20—C19	116.9 (3)	C45—C44—H44	120.3
O3—C20—C21	123.1 (3)	C44—C45—H45	120.1
C19—C20—C21	120.0 (4)	C44—C45—C46	119.8 (4)
C16—C21—C20	119.4 (3)	C46—C45—H45	120.1
C16—C21—H21	120.3	C45—C46—H46	119.9
C20—C21—H21	120.3	C47—C46—C45	120.2 (4)
O3—C22—H22A	108.7	C47—C46—H46	119.9
O3—C22—H22B	108.7	C46—C47—H47	119.7
O3—C22—C23	114.1 (3)	C46—C47—C48	120.6 (4)
H22A—C22—H22B	107.6	C48—C47—H47	119.7
C23—C22—H22A	108.7	C43—C48—C47	118.3 (4)
C23—C22—H22B	108.7	C43—C48—H48	120.8
C24—C23—C22	121.4 (3)	C47—C48—H48	120.8
			
O2—C16—C17—C18	178.9 (3)	C19—C20—C21—C16	0.1 (5)
O2—C16—C21—C20	−178.5 (3)	C20—O3—C22—C23	−80.0 (4)
O3—C20—C21—C16	179.4 (3)	C21—C16—C17—C18	0.5 (6)
O3—C22—C23—C24	−30.7 (5)	C22—O3—C20—C19	−165.2 (3)
O3—C22—C23—C28	152.4 (3)	C22—O3—C20—C21	15.4 (5)
O4—C29—C30—C31	−179.6 (4)	C22—C23—C24—C25	−174.6 (3)
O4—C29—C30—C32	−3.9 (7)	C22—C23—C28—C27	174.6 (3)
O5—C37—C38—C39	179.1 (3)	C23—C24—C25—C26	−0.1 (5)
O5—C37—C42—C41	−178.6 (3)	C23—C24—C25—C29	174.4 (3)
O6—C1—C2—C3	−165.4 (3)	C24—C23—C28—C27	−2.3 (5)
O6—C1—C2—C7	12.6 (5)	C24—C25—C26—C27	−1.9 (5)
O6—C41—C42—C37	179.0 (3)	C24—C25—C26—C31	174.7 (3)
C1—O6—C41—C40	−160.6 (3)	C24—C25—C29—O4	4.5 (6)
C1—O6—C41—C42	19.3 (5)	C24—C25—C29—C30	−173.6 (4)
C1—C2—C3—C4	175.2 (4)	C25—C26—C27—C28	1.8 (5)
C1—C2—C7—C6	−175.9 (3)	C25—C26—C31—C30	0.0 (4)
C2—C3—C4—C5	0.5 (6)	C25—C26—C31—C35	−177.1 (4)
C3—C2—C7—C6	2.1 (5)	C25—C29—C30—C31	−1.5 (4)
C3—C4—C5—C6	2.3 (5)	C25—C29—C30—C32	174.2 (4)
C3—C4—C5—C8	−172.2 (4)	C26—C25—C29—O4	179.6 (4)
C4—C5—C6—C7	−3.1 (5)	C26—C25—C29—C30	1.5 (4)
C4—C5—C6—C10	−179.0 (3)	C26—C27—C28—C23	0.3 (6)
C4—C5—C8—C9	174.1 (4)	C26—C31—C35—C34	174.0 (4)
C4—C5—C8—C11	−3.6 (7)	C27—C26—C31—C30	176.1 (4)
C5—C6—C7—C2	0.8 (5)	C27—C26—C31—C35	−1.0 (7)
C5—C6—C10—O1	−173.4 (4)	C28—C23—C24—C25	2.2 (5)
C5—C6—C10—C9	6.1 (4)	C29—C25—C26—C27	−177.5 (3)
C5—C8—C9—C10	4.8 (4)	C29—C25—C26—C31	−0.9 (4)
C5—C8—C9—C14	−172.1 (3)	C29—C30—C31—C26	1.0 (4)
C5—C8—C11—C12	173.4 (4)	C29—C30—C31—C35	178.4 (3)
C6—C5—C8—C9	−0.9 (4)	C29—C30—C32—C33	−174.1 (4)
C6—C5—C8—C11	−178.6 (4)	C30—C31—C35—C34	−2.8 (5)
C7—C2—C3—C4	−2.9 (5)	C30—C32—C33—C34	−4.0 (5)
C7—C6—C10—O1	11.1 (6)	C30—C32—C33—C36	171.1 (3)
C7—C6—C10—C9	−169.4 (4)	C31—C26—C27—C28	−173.9 (4)
C8—C5—C6—C7	172.6 (3)	C31—C30—C32—C33	1.2 (5)
C8—C5—C6—C10	−3.3 (4)	C32—C30—C31—C26	−175.2 (3)
C8—C9—C10—O1	172.7 (4)	C32—C30—C31—C35	2.3 (5)
C8—C9—C10—C6	−6.7 (4)	C32—C33—C34—C35	3.5 (5)
C8—C9—C14—C13	−2.0 (5)	C32—C33—C36—O5	33.9 (5)
C8—C11—C12—C13	−1.6 (6)	C33—C34—C35—C31	0.0 (6)
C9—C8—C11—C12	−4.0 (5)	C34—C33—C36—O5	−151.0 (3)
C10—C6—C7—C2	175.7 (3)	C36—O5—C37—C38	167.2 (3)
C10—C9—C14—C13	−178.2 (4)	C36—O5—C37—C42	−13.0 (5)
C11—C8—C9—C10	−177.2 (3)	C36—C33—C34—C35	−171.7 (3)
C11—C8—C9—C14	5.9 (6)	C37—O5—C36—C33	78.4 (4)
C11—C12—C13—C14	5.5 (6)	C37—C38—C39—C40	0.1 (6)
C11—C12—C13—C15	−170.5 (4)	C38—C37—C42—C41	1.3 (5)
C12—C13—C14—C9	−3.6 (5)	C38—C39—C40—C41	0.0 (6)
C12—C13—C15—O2	168.8 (3)	C39—C40—C41—O6	−179.6 (3)
C14—C9—C10—O1	−10.7 (7)	C39—C40—C41—C42	0.5 (5)
C14—C9—C10—C6	169.9 (4)	C40—C41—C42—C37	−1.2 (5)
C14—C13—C15—O2	−7.0 (5)	C41—O6—C1—C2	−90.6 (4)
C15—O2—C16—C17	158.0 (3)	C42—C37—C38—C39	−0.7 (5)
C15—O2—C16—C21	−23.6 (5)	Cl1—C43—C44—C45	−177.9 (3)
C15—C13—C14—C9	172.1 (3)	Cl1—C43—C48—C47	177.0 (4)
C16—O2—C15—C13	91.6 (4)	C43—C44—C45—C46	0.5 (6)
C16—C17—C18—C19	−0.7 (6)	C44—C43—C48—C47	−2.2 (7)
C17—C16—C21—C20	−0.2 (6)	C44—C45—C46—C47	−1.4 (7)
C17—C18—C19—C20	0.7 (6)	C45—C46—C47—C48	0.5 (7)
C18—C19—C20—O3	−179.7 (3)	C46—C47—C48—C43	1.3 (7)
C18—C19—C20—C21	−0.3 (6)	C48—C43—C44—C45	1.3 (7)

### Hydrogen-bond geometry (Å, º)

*Cg*1, *Cg*2 and *Cg*15 are the centroids of the C5/C6/C10/C9/C8, C25/C29/C30/C31/C26 and C43–C48 rings, respectively.

**Table:**

*D*—H···*A*	*D*—H	H···*A*	*D*···*A*	*D*—H···*A*
C18—H18···Cl1^i^	0.95	2.83	3.547 (4)	133
C35—H35···O1^ii^	0.95	2.58	3.491 (5)	161
C46—H46···O6^iii^	0.95	2.55	3.418 (5)	152
C1—H1*A*···*Cg*2^iv^	0.99	2.95	3.610 (4)	125
C22—H22*A*···*Cg*1^v^	0.99	2.73	3.711 (4)	170
C36—H36*B*···*Cg*15^vi^	0.99	2.84	3.713 (4)	148

Symmetry codes: (i) *x*+1, *y*+1, *z*; (ii) *x*−1, *y*, *z*; (iii) *x*+1, *y*+1, *z*+1; (iv) *x*, *y*−1, *z*; (v) *x*, *y*+1, *z*; (vi) *x*−1, *y*, *z*−1.
